# Supplementary material for: A protocol for using rapid qualitative techniques to incorporate multi-level stakeholder feedback in a pragmatic clinical trial of mindfulness for chronic low back pain
Source: PLoS One. 2026 Jan 2;21(1):e0338304. doi: 10.1371/journal.pone.0338304 (PMC12758753; doi:10.1371/journal.pone.0338304)
Supplement: S1 Appendix — Appendix A: IRB Approved Protocol Language. The following section contains language approved by the University of Pittsburgh’s Institutional Review Board for the Stakeholder Engagement Protocol. Appendix B: Interview and Focus Group Guides. The following section contains semi-structured interview and focus group guides for the qualitative portions of the Stakeholder Engagement Protocol. (DOCX) [file pone.0338304.s001.docx]

# Appendix A: IRB Approved Protocol Language

**Pitt IRB#** STUDY20110378

STAKEHOLDER ENGAGEMENT
 Future successful implementation of the Mindfulness-based Stress Reduction (MBSR) program in primary care healthcare settings requires the perspectives of the people involved. These stakeholder opinions will help to describe barriers and facilitators to integrating MBSR into primary care. These stakeholder perspectives will assure that we address integration issues that as scientists we would not otherwise be aware of. We will conduct individual interviews of clinic staff, healthcare system professionals, and payors (insurance representatives). We will also conduct focus groups of OPTIMUM Study participants who are all persons with chronic low back pain.

1. Individual interviews: Individuals such as clinic staff (front desk/registration, nurses, nurse practitioners, medical assistants, clinic managers, medical directors), healthcare system professionals (medical staff directors, providers, nursing staff directors, clinic and health system administrators), and payors (insurance representatives) will be providing their expert opinions in one-on-one interviews with trained study personnel. These individuals will undergo verbal informed consent. The verbal informed consent form has been uploaded and it is titled “Verbal individual interview consent form.” Up to 60-minute individual interviews will be collected via phone or videoconference. The interviews will be recorded and transcribed and analyzed for common themes. Participants will be reimbursed $100 for the interview.

2. Focus groups: OPTIMUM study participants who are all persons with chronic low back pain will be invited to participate in a focus group about their experiences as a research participant. Verbal informed consent will be obtained in one of two ways: a. If the participant was consented prior to the November 2021 informed consent modification, then they will undergo an addendum verbal consent, this consent is titled “Addendum focus group consent form.” b. The informed consent after November 2021 now contains the language about participating in the focus group. The interviews will be between 1 to 2 hours long and will be collected via videoconference, recorded and transcribed, and analyzed for common themes. Participants will be reimbursed $100 for their participation in the focus group.

APPENDIX B:

Interview and Focus Group Guides

*These are semi-structured interview guides and are intended to guide the conversation, though actual interviews may include additional probes, requests for clarification, or rephrasing.*

Thank you for agreeing to participate in this interview. The purpose of this interview is to understand your experiences with research, and in particular, with the OPTIMUM study. Your feedback will be used to help improve the ongoing study, and to inform future research.

We are asking for your permission to audio record this interview using this secure zoom functionality. Recording this interview well ensure we do not miss any of your comments. If you agree, the audio recording will be stored on a secure, password protected server and will be transcribed without your name. Only members of the research team will have access to your comments, and any reports including your comments will never use your name. You can ask for the recording to be stopped at any time. After the reports are written the audio recording will be destroyed at the completion of the study.

Do I have your permission to record this interview?

[If yes] Thank you for giving me permission to record. I have started the recording now.

Please keep in mind we are asking these questions so that we can improve the program. We would like your honest feedback, positive or negative, as our goal is to improve.

Participant Focus Group Guide:

1. Please tell us about your experience participating in research studies.
2. Who has participated in research studies before?
3. How do you hear about research studies?
4. How do you decide to participate in a research study?
5. Why have you decided to participate in research studies in the past?
6. Please tell us about your experience so far with the OPTIMUM trial.
7. How did you learn about the OPTIMUM study?
8. What caught your attention about the OPTIMUM study?
9. What was the reason you decided to participate in the OPTIMUM study?
10. What ways do you prefer to be contacted about research studies?
11. What do you want to know or understand about a study when you’re deciding whether or not to participate?
12. What are your ideas about how we could recruit more patients to participate in the OPTIMUM study?
13. What do you think is working really well with the OPTIMUM study?
14. What do you think could be improved with the OPTIMUM study?
15. We are trying to find ways to keep participants motivated to participate in the study for a full year. What are things that motivate you to continue to participate in OPTIMUM?
16. What is working about what the research team is currently doing to keep you engaged?
17. What are ways the research team could do a better job of keeping you engaged?
18. Sometimes it can be difficult to reach participants. Do you have suggestions for how we can reach participants more successfully?
19. Is there anything else you’d like to add? Anything I haven’t asked you that I should have asked?

Insurers:

Introduction: Mindfulness-based Stress Reduction (MBSR) is now recommended as a first line therapy for patients with chronic low back pain by the American College of Physicians (the professional organization for general internal medicine physicians).  We (I?) would like to understand how your insurance company makes decisions to cover new therapies like MBSR for patients.


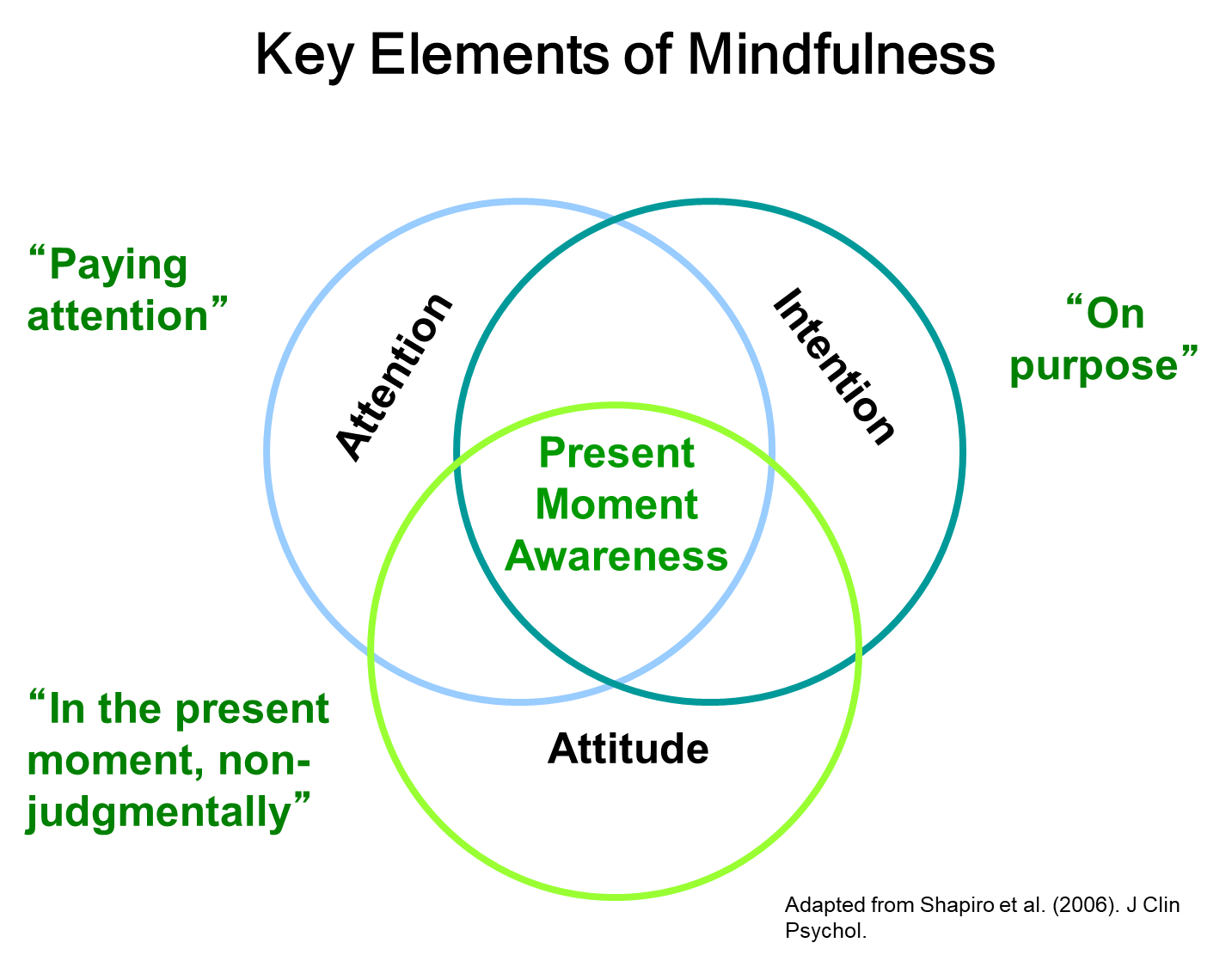


1. Tell me about your professional role—what do you do?
2. Currently MBSR is not covered by insurance. How does a decision get made to have an evidenced based therapy for chronic low back pain like MBSR covered by insurance?
3. What are the barriers for covering this therapy in an insurance plan?
4. What outcomes influence your decision to cover a therapy like this?
5. What types of cost outcomes do you look at?
6. Are there other outcomes you look at for therapies like these (ie acunpuncture or chiropractic)?
7. MBSR could be offered as a telehealth or video group medical visit with a mindfulness instructor and provider both participating. What do you think of this model?


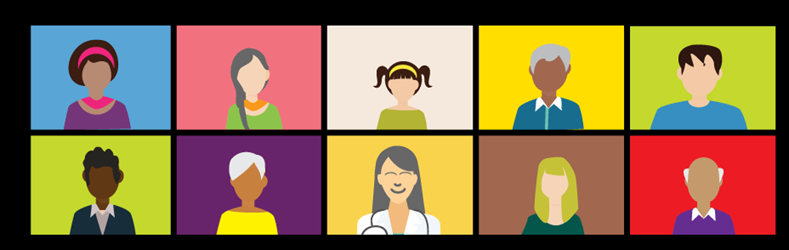
Image Credit: Zannie Gunn, Graphic Designer, University of North Carolina at Chapel Hill


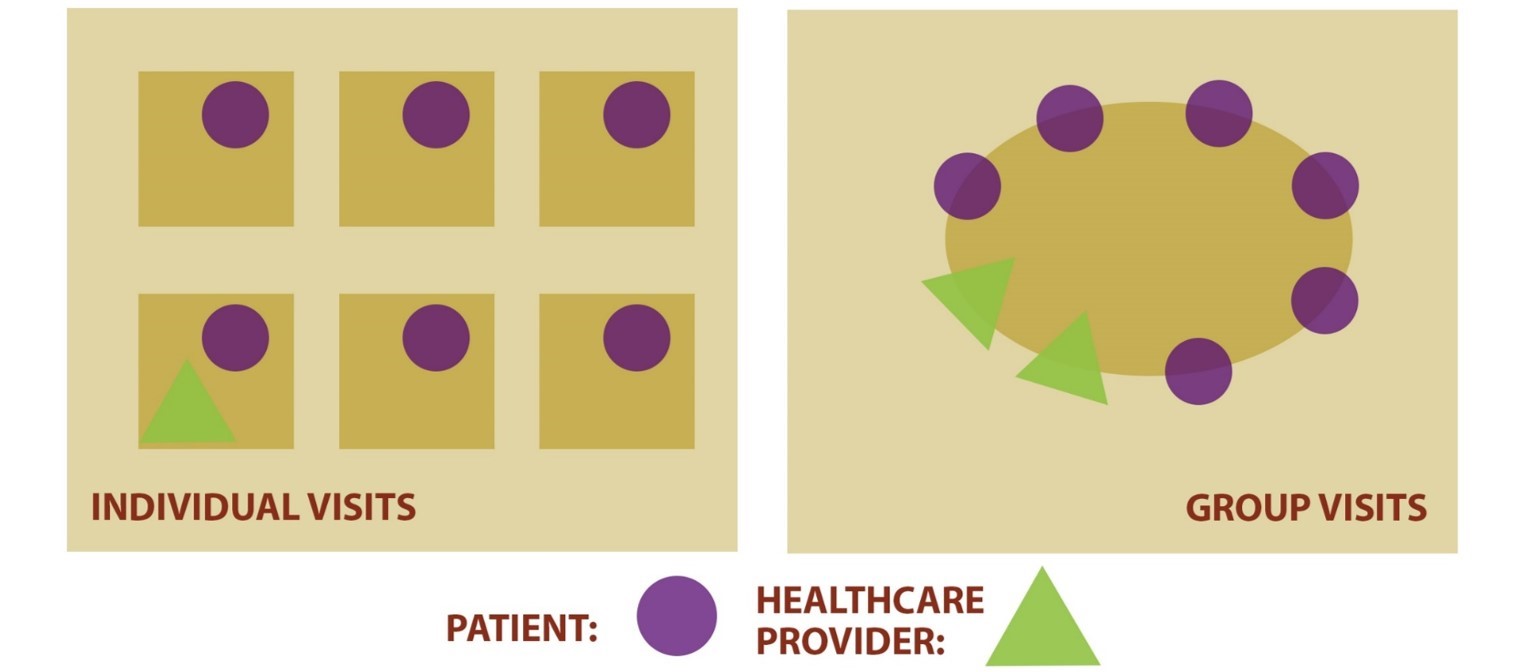
Image Credit: Dr. Ariana Thompson-Lastad, University of California San Francisco: Rachel Schragis, Graphic Designer

1. Group medical visits are where one or two providers or co facilitators see multiple patients with a common condition simultaneously
2. Tell me, are telehealth medical visits covered? Why or why not?
3. Is there anything else you’d like to add? Anything I haven’t asked you that I should have asked?

Providers:

1. Tell me about your professional role—what do you do?
2. Tell me about your experience treating patients with chronic low back pain.
3. Mindfulness-based stress reduction is an evidence based group therapy for chronic low back pain which is currently not widely offered in primary care. What do you know about MBSR?


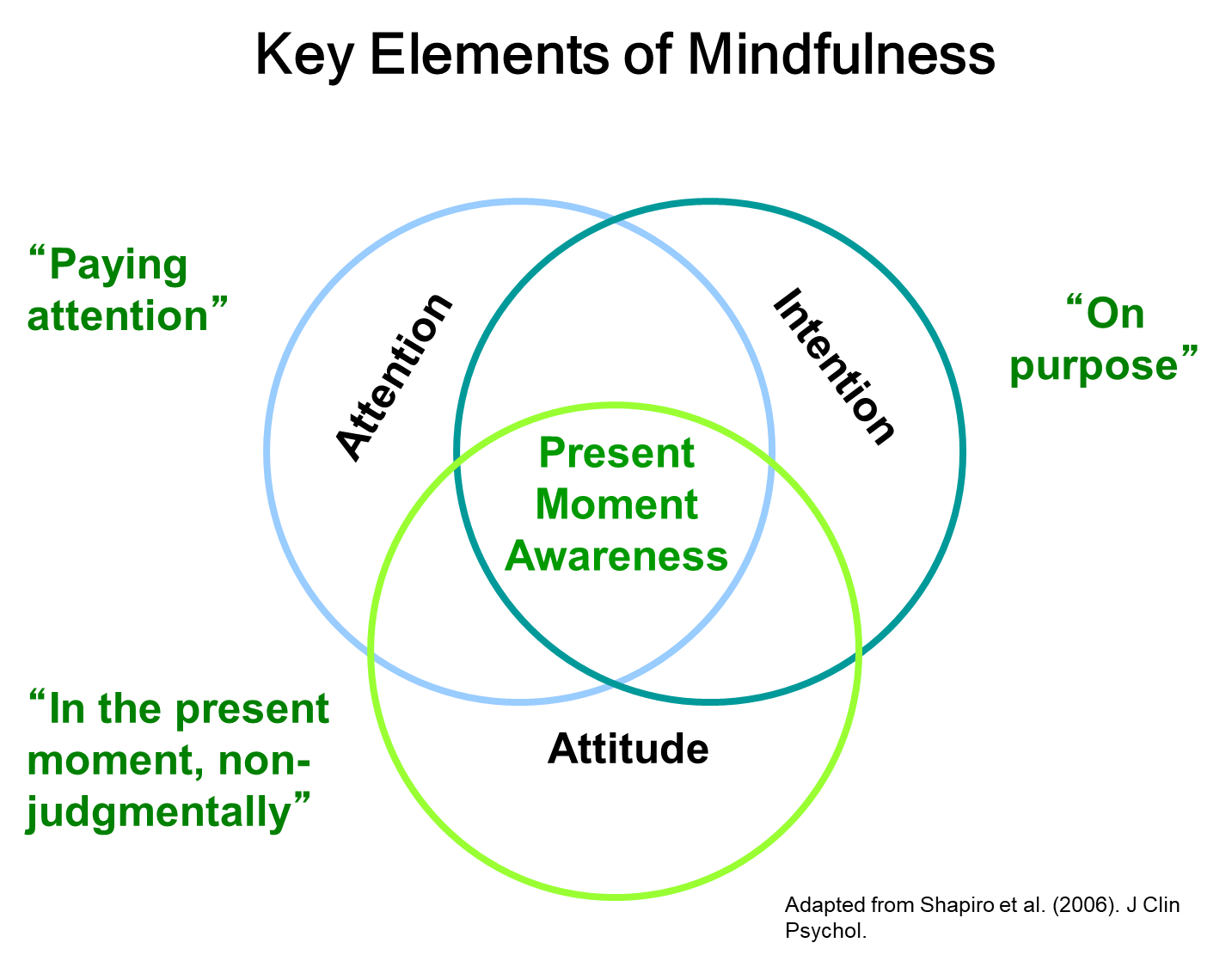


1. What challenges do you foresee with integrating mindfulness into the clinic?
2. How could those challenges be overcome?
3. What do you think needs to happen to successfully integrate mindfulness into the clinic?
4. Who needs to get on board with implementation to successfully integrate mindfulness into the clinic?
5. MBSR could be offered as a telehealth or video group medical visit with a mindfulness instructor and provider both participating. What do you think of this model? Insert model of a group medical visit. Group medical visits are where one or two providers or co facilitators see multiple patients with a common condition simultaneoulsly
6. Tell me, what you know about group medical visits?
7. Would you be interested in seeing patients as part of a group medical visit? Why or why not
8. Would you be interested in seeing patients as part of a telehealth or video group medical visit? Why or why not

These next few questions are about research studies

1. Tell me about your general experience referring your patients to research studies.
2. In your experience, what are the most successful ways studies can recruit your patients to participate in research?
3. Can you describe a study that used successful recruitment methods with your patients?  What happened? What methods were used? What did not work?
4. Who were the key clinic people involved?
5. What made this experience successful?
6. In your experience, what are the least successful ways, or barriers encountered by studies recruiting your patients?
7. Please tell me about your experience so far with the OPTIMUM trial.
8. [IF NONE—then what? Describe using visuals? Skip questions that are N/A?]
9. How did you learn about the study?
10. OPTIMUM is recruiting for three years.  What recruitment methods are you aware of?
11. What ways, if any, would you like to be reminded or prompted to refer patients?
12. What helps you to be comfortable in referring a patient?
13. What makes you uncomfortable making a referral?
14. What opinions, if any, do you have about current study recruitment methods?
15. What challenges, if any, have you observed with current recruitment methods?
16. What do you think could be done to recruit participants into the study?
17. Probe: What do you think would be feasible [at your site]?
18. What do you think are the best ways to keep providers reminded about OPTIMUM?
19. In what ways, if any, would you like information about the status of a patient you have referred to OPTIMUM?
20. In what ways, if any, would you like information about OPTIMUM study results?
21. Is there anything else you’d like to add? Anything I haven’t asked you that I should have asked?

Administrative Stakeholders:

1. Tell me about your professional role—what do you do?
2. What’s your title?
3. What professional experience do you have working with research teams at your organization (not personal role as a research participant)?
4. If contributed to a research study, then probe:
5. Tell me about your experience with a study
6. What was your role?
7. How did you engage with the research?
8. In your experience working with research teams, what has worked well?
9. What was challenging?
10. What are some methods that you believe would make patients likely to participate in research studies?

The intervention we are testing is delivered via telehealth.

1. Do you currently offer telehealth visits?

If yes...

1. What has that been like providing telehealth in your clinic?
2. What’s been successful?
3. What’s been challenging?
4. Do you think you will continue offering telehealth services?

If no...

1. Why not?
2. Do you currently offer group telehealth visits?

If yes...

1. What has that been like providing group telehealth in your clinic?
2. What’s been successful?
3. What’s been challenging?
4. Do you think you will continue offering group telehealth services?

If no...

1. Why not?
2. Mindfulness-based stress reduction (MBSR) is an evidence-based therapy for chronic low back pain which is currently not widely offered in primary care. MBSR could be offered as a telehealth group medical visit with mindfulness instructor and provider both participating. What do you think of this model?
3. What would some of the benefits be of offering this model to patients in your clinic?
4. What could some of the barriers be for offering this model in your clinic?
5. How do you think some of those barriers could be overcome?

1. What would need to happen for an MBSR instructor to be employed at your organization and co-leading the telehealth group visits with a provider?
2. What would the process be to credential mindfulness instructors to teach mindfulness in your (the) health care system?
3. How might someone without other medical credentials be credentialed in your organization?
4. Are there other similar roles in your clinic already?
5. If they have no idea...who would know? Who should be ask?
6. Is there anything else you’d like to add? Anything I haven’t asked you that I should have asked?
