# Supplementary material for: A protocol for using rapid qualitative techniques to incorporate multi-level stakeholder feedback in a pragmatic clinical trial of mindfulness for chronic low back pain
Source: PLoS One. 2026 Jan 2;21(1):e0338304. doi: 10.1371/journal.pone.0338304 (PMC12758753; doi:10.1371/journal.pone.0338304)
Supplement: Attachment — Submitted filename: A Protocol for Using Rapid Qualitative Techniques.docx [file pone.0338304.s002.docx]

A Protocol for Using Rapid Qualitative Techniques to Incorporate Multi-Level Stakeholder Feedback in a Pragmatic Clinical Trial of Mindfulness for Chronic Low Back Pain

Outstanding study and well done. This paper has a potential to be accepted, but some important points have to be clarified or fixed before we can proceed and a positive action can be taken. Given these shortcomings the manuscript requires major revisions. I have some suggestions for each aspect of the manuscript. We here summarize these points:

**Abstract**:

Please focus the introduction in the abstract on the current study and your aims. The introduction is lengthy, and the last two lines are unnecessary and vague. In the methodology should be summarized, emphasizing the sample of this study and the methods of data collection.

The conclusion provides a clear and structured response to the main question or Purpose of the current research. Please conclude in relation to the overall Purpose or main question of the study and avoid generalizations.

**Introduction:**

In the introduction section authors should begin with a general context, narrowing to the specific focus of the manuscript. Include answer five main question: why your research is important, what is already known about the topic, the “gap” or what is not yet known about the topic, why it is important to learn the new information that your research adds, and the specific research aim(s) that your manuscript addresses. Your research aim should address the gap you identified. Be sure to add enough background information to enable readers to understand your study. Please clearly demonstrate the research problem of the present study from both a tactical (theoretical necessity) and a content (practical necessity) perspective.

In the introduction, more attention should be given to background information, research problem, research objective(s) and research question, making them tangible and understandable for the reader.

**Methodology:**

There is very little info on the development of this protocol (details, implementation, fidelity). The explanations are general; please address the details of the design and feasibility of the protocol.

It is recommended to evaluate the psychometric characteristics of the designed protocol in this study based on the principles of developing therapeutic interventions according to the American Psychological Association’s guidelines for assessing treatment protocols in three areas: the process of developing guidelines, Treatment Efficacy, and Clinical Utility.

The first criterion in the process of developing guidelines pertains to the members of the intervention development working group. What areas of expertise did the working group for the designed protocol include?

As the second criterion, the ethical evaluation of the developed program was conducted by whom?

The evaluation of treatment protocols by the American Psychological Association in the section on the therapeutic effectiveness of the intervention includes five criteria. The first criterion pertains to the theoretical foundations of the designed intervention. The second criterion is based on scientific clinical observations and the consensus of experts regarding the intervention topic. The third criterion addresses the effectiveness of the intervention in terms of the desirability of outcomes both exclusively and in comparison, to other related interventions. The fourth criterion in the area of treatment effectiveness relates to the alignment between the treatment and the patient. In the fifth criterion, provide the necessary documentation to specify the outcomes of the intervention and present supporting evidence.

what did the sessions involve, who delivered the sessions, who was present during the session, what format were the sessions delivered in (face-to-face, website, workbooks?), where were the sessions delivered (clinic, home?) A table with the topics of each session is not enough.

**Results:**

How were the psychometric characteristics of the current protocol examined? How many members were in the focus group, and what specialties did they have? How was the content of the interviews coded? What software was used for coding? Please provide the necessary explanations on these matters.

Qualitative research software should be used for coding interviews

**Discussions:**

in the discussion should beginning with interpretation of your results and moving to general implications. begins with a restatement of the main findings, which can usually be accomplished with a few carefully-crafted sentences. Next, interpret the meaning or explain the significance of your results, lifting the reader’s gaze from the study’s specific findings to more general applications. Then, compare these study findings with other research. Are these findings in agreement or disagreement with those from other studies? Does this study impart additional nuance to well-accepted theories? Situate your findings within the broader context of scientific literature, then explain the pathways or mechanisms that might give rise to, or explain, the results. Keep in mind that every study has strengths and limitations. Candidly reporting yours helps readers to correctly interpret your research findings. The next element of the discussion is a summary of the potential impacts and applications of the research. Should these results be used to optimally design an intervention? Does the work have implications for clinical protocols or public policy? These considerations will help the reader to further grasp the possible impacts of the presented work. Finally, the discussion should conclude with specific suggestions for future work. Here, you have an opportunity to illuminate specific gaps in the literature that compel further study. Avoid the phrase “future research is necessary” because the recommendation is too general to be helpful to readers. Instead, provide substantive and specific recommendations for future studies.

**Conclusions**:

Please provide a clear, objective, and tangible response to the overall research question and the feasibility of the current protocol for the target population.

References:

Please use more recent and high-quality references.

The text of the article needs to be simplified and made more fluid for better communication with the reader. Therefore, it is suggested that the authors simplify, trim, and summarize some of the long and unnecessary sentences to keep the reader’s mind aligned with the flow of the study’s progression.
