# Supplementary material for: A protocol for using rapid qualitative techniques to incorporate multi-level stakeholder feedback in a pragmatic clinical trial of mindfulness for chronic low back pain
Source: PLoS One. 2026 Jan 2;21(1):e0338304. doi: 10.1371/journal.pone.0338304 (PMC12758753; doi:10.1371/journal.pone.0338304)
Supplement: Attachment — Submitted filename: Response to Reviewers.6.1.25.docx [file pone.0338304.s005.docx]

| *Reviewer #1:*  Thank you for responding to the reviewers' comments. However, some unresolved issues remain.  I cannot locate Appendix B in the revision.  What new references about rapid qualitative methods were added to the revised manuscript?  If policy influencer and advocacy groups are used synonymously throughout the paper, it would be beneficial to use only one of those terms. | Thank you for this comment.  Appendix B was previously attached as a separate document, we have also added Appendix B to the end of the manuscript file for ease of review for this submission.  New references about rapid qualitative methods include:  [8] R. E. Keith, J. C. Crosson, A. S. O’Malley, D. Cromp, and E. F. Taylor, “Using the Consolidated Framework for Implementation Research (CFIR) to produce actionable findings: a rapid-cycle evaluation approach to improving implementation.,” *Implement. Sci.*, vol. 12, no. 1, p. 15, Feb. 2017, doi: 10.1186/s13012-017-0550-7.  [9] C. Vindrola-Padros and G. A. Johnson, “Rapid techniques in qualitative research: A critical review of the literature.,” *Qual. Health Res.*, vol. 30, no. 10, pp. 1596–1604, Aug. 2020, doi: 10.1177/1049732320921835.  [10] B. Taylor, C. Henshall, S. Kenyon, I. Litchfield, and S. Greenfield, “Can rapid approaches to qualitative analysis deliver timely, valid findings to clinical leaders? A mixed methods study comparing rapid and thematic analysis.,” *BMJ Open*, vol. 8, no. 10, p. e019993, Oct. 2018, doi: 10.1136/bmjopen-2017-019993.  [11] A. L. Nevedal *et al.*, “Rapid versus traditional qualitative analysis using the Consolidated Framework for Implementation Research (CFIR).,” *Implement. Sci.*, vol. 16, no. 1, p. 67, Jul. 2021, doi: 10.1186/s13012-021-01111-5.  We have changed the terminology throughout the document to refer to ‘policy advocates’ |
| --- | --- |
| Reviewer #2: I agree with the reviewer #1 :”Although the authors acknowledge the need for more research and data on the stakeholder engagement process for integrative medicine, it is less clear how this protocol will address this knowledge gap.  Given the parent study is concluding this calendar year, I have some concerns about the lack of details provided by the authors if they do not get adequate representation from patients and stakeholders in the focus groups and interviews they plan to conduct. The emphasis on snowball sampling may be the most appropriate option in this supplement study. Still, there are potential risks that the authors should acknowledge if they do not reach thematic saturation. Specifically, suppose the focus is to enhance the representation of underrepresented patients in the parent study. In that case, it is unclear how the authors will be able to recruit underrepresented patients for the supplement study.” | Thank you for this comment. We have added additional references about rapid qualitative methods being useful for incorporating stakeholder input, as well as explaining that rapid qualitative methods allow us to incorporate feedback in real time (lines 59-60).  We added additional detail on the limitations of our sampling strategies and potential for not reaching thematic saturation in the discussion section in our previous revision, based on reviewer 1’s feedback (lines 257-271). Unfortunately, due to the timing of the review process, we are not able to change the methodology at this time.  The focus is to enhance representation of patient voices into the parent trial, therefore, the representation of patients will be inherently limited by the recruited patients through the parent trial. |
