# Supplementary material for: A protocol for using rapid qualitative techniques to incorporate multi-level stakeholder feedback in a pragmatic clinical trial of mindfulness for chronic low back pain
Source: PLoS One. 2026 Jan 2;21(1):e0338304. doi: 10.1371/journal.pone.0338304 (PMC12758753; doi:10.1371/journal.pone.0338304)
Supplement: Attachment — Submitted filename: Response to Reviewers.docx [file pone.0338304.s006.docx]

## Response to Reviewers: September 15, 2025

REVIEWER: It is unclear how the authors will be able to recruit underrepresented patients for
the supplement study.

RESPONSE: We have included a detailed explanation of our sampling strategy to ensure a diverse sample of underrepresented demographic groups on page 7, lines 151-158:

“To ensure that a diverse sample of underrepresented patients are represented in the focus groups in the supplement study, we will first evaluate the reported demographic factors of all potential participants. A random number generator will be used to select 10 individuals, followed by an assessment to ensure that the group reflects the diversity of the parent study population. Participants are then assigned a number attached to their participant ID and the generator will randomly select a number. The research team will then contact the participant to invite them to participate and if a selected participant is unavailable, another demographically similar participant was contacted to maintain group diversity.“

REVIEWER: Here are potential risks that the authors should acknowledge if they do not reach thematic saturation

RESPONSE: To address risks of not reaching thematic saturation, we have modified the methods on page 7, lines 147-149 and page 8, lines 172-173 to specify that data collection will continue until thematic saturation is reached. We have also acknowledged risks of not reaching thematic saturation in the discussion section on page 15, lines 329-331 and explained how we are mitigating this risk.
